# Vertical Cavity Surface Emitting Laser Performance Maturing through Machine Learning for High-Yield Optical Wireless Network

**DOI:** 10.3390/mi13122132

**Published:** 2022-12-01

**Authors:** Ammar Armghan, Khaled Aliqab, Farman Ali, Fayadh Alenezi, Meshari Alsharari

**Affiliations:** 1 Department of Electrical Engineering, College of Engineering, Jouf University, Sakaka 72388, Saudi Arabia; 2 Department of Electrical Engineering, Qurtuba University of Science and IT, Dera Ismail Khan 29050, Pakistan

**Keywords:** optical wireless network, vertical cavity surface emitting laser, machine learning, multiple users with high data transmission, mathematical approach

## Abstract

The high-yield optical wireless network (OWN) is a promising framework to strengthen 5G and 6G mobility. In addition, high direction and narrow bandwidth-based laser beams are enormously noteworthy for high data transmission over standard optical fibers. Therefore, in this paper, the performance of a vertical cavity surface emitting laser (VCSEL) is evaluated using the machine learning (ML) technique, aiming to purify the optical beam and enable OWN to support high-speed, multi-user data transmission. The ML technique is applied on a designed VCSEL array to optimize paths for DC injection, AC signal modulation, and multiple-user transmission. The mathematical model of VCSEL narrow beam, OWN, and energy loss through nonlinear interference in an optical wireless network is studied. In addition, the mathematical model is then affirmed with a simulation model following the bit error rate (BER), the laser power, the current, and the fiber-length performance matrices. The results estimations declare that the presented methodology offers a narrow beam of VCSEL, mitigating nonlinear interference in OWN and increasing energy efficiency.

## 1. Introduction

An optical wireless network (OCN) is a delicate structure to accommodate 5G and 6G services and fulfil high data transmission demands. However, the high order losses such as diffraction and nonlinear interference are the focused parameters that add extra unwanted frequencies to the original channel frequencies and thus result in 5G and 6G services that can be interrupted [1,2,3]. High-brightness, high-speed, and narrow spectral-light-source-based channels are considered low-cost and optimum solutions to overcome the issues discussed above. The narrow spectral channel transmission is currently possible by applying the vertical cavity surface emitting lasers (VCSELs) in OWN [4]. The VCSEL array produces high-brightness and single mode outputs with a narrow divergence angle, which supports multi-channel transmission. However, on the other side, the peak relative intensity noise (RIN), low-frequency roll off in the electrical to optical (O/E) frequency response, and the low-photon lifetime are the significant matters that can enhance nonlinear channel interference and confined OWN space for 5G and 6G services [5,6]. Therefore, to achieve mature narrow efficient outputs from the VCSEL array, the issues mentioned earlier must be addressed. Recently, advanced machine learning (ML) techniques have great potential for model optimization, as well as low-profile-based circuits [7,8]. Hence, in this work, we evaluate the ML methodologies, aiming to gain a coherent VCSEL yield for high-speed channels in OWN.

### 1.1. Prior Work

Many efforts have been made on OWN and VCSEL array performance advancement to accommodate the following generation services. For enhanced OWN and multi-channel transmission coverage, a novel quasi single mode VCSEL (QSM-VCSEL) is designed in [9], which exhibits a flatter E/O response and narrower divergence angle. In another paper [10], the QSM-VCSEL array is introduced in terms of electrodes and Zn-diffusion apertures, to develop high-yield data transmission in OWN. The authors in [11] discussed deep neural networks to improve the O/E conversion speed in the VCSEL-based transmitter array. Paper [12] investigated the zero-forcing technique, aiming to manage multi-user interference in the VCSEL-transmitter-based OWN, which has enhanced throughput and energy efficiency. In addition, mixed integer linear programming (MILP) and Q-learning mechanisms are applied on VCSELs based on OWN [13], where challenges such as resource allocation are investigated. To be precise, regarding the outputs of the VCSEL array for high data transmission in OWN, an analogue coherent is proposed in [14]. In [15], the authors have presented a coherent optical coupling methodology for the VCSEL array, which has developed the transmitter’s bandwidth. The study in [16] demonstrated a 940 nm VCSEL array to obtain high speed efficiency and high-brightness outputs. The authors in [17] described a new OWN-based VCSEL to compensate for nonlinear channel interference. Similarly, in [18] and [19], the noise level, frequency response, and nonlinear interference are considered and showed that, owing to VCSEL’s narrow bandwidth, high energy density and high-speed features can optimize the OWN structure and take into account the noise level, low-frequency response, and nonlinear interference. The above studies conclude that numerous pieces of research have been conducted on boosting OWN-based VCSEL executions. However, these suggested ways cannot be adopted to endorse 6G jobs and oblige multi-channel transmission, including online and live video demands. Furthermore, currently the OWN is widely used for non-stationary objects such as unmanned aerial vehicles (UAV) to ground communication [20], which requires amendments to ongoing OWN-based VCSEL array transmitters.

### 1.2. Motivation

The advancement in OWN is vital for the present 5G and next 6G services, which can be made possible by applying a VCSEL-array-based transmitter. In addition, the narrow-spectral-width-based channel transmission in OWN is mainly required for non-stationary and aerial communication such as UAV to ground communication systems. The suggested VCSEL models for OWN in the literature still include noise level, DC-injection and AC-signal-modulation accuracy, low-frequency response, and maximum RIN. It is also noted that for next-generation OWN, the current approaches are limited to supporting high transmission speed and multi-channel transmission with increased coverage area. The enormous features of VCSEL-based OWN motivated us to extract the characteristics of the VCSEL array, upgrade the reliability of OWN, and handle the next generation 6G and UAV communication services.

### 1.3. Main Contribution

In order to obtain the OWN results and increase the transmission speed, including capacity, in this paper, the ML-based VCSEL structure is proposed. The high power OWN is the main requirement for today’s 5G and 6G services. The significant contributions of this paper are summarized as follows.

1. High-brightness and high-speed VCSEL are evaluated using the ML algorithm, to purify the optical beam and enable the OWN to handle high-speed data transmission.
2. The transmitter side’s digital signal processing (DSP) block is designed for OWN using the advanced quadrature amplitude modulation-orthogonal frequency division multiplexing (QAM-OFDM) scheme. This technique has strengthened the OWN outputs against novel impairments such as nonlinearities.
3. Various new procedures are applied, examining OWN and presenting ML-based VCSEL efficiencies such as data rate, spectral efficiency (SE), power levels, fiber path cover, and different visualizers.
4. The ML mode is evaluated for the presented VCSEL, and a detailed analytical model is discussed and matched with the simulation estimations.
5. The simulation results are measured and compared to the achievements of the proposed ML-based VCSEL and OWN with conventional laser sources.

The rest part of the manuscript is organized as follows: the VCSEL-based OWN model is presented in Section 2, Section 3 consists of the results analysis of the presented ML-based VCSEL and the high-capacity data rate OWN, and the conclusion of the paper is given in Section 4.

## 2. VCSEL-Based OWN System Model

**Figure 1:**
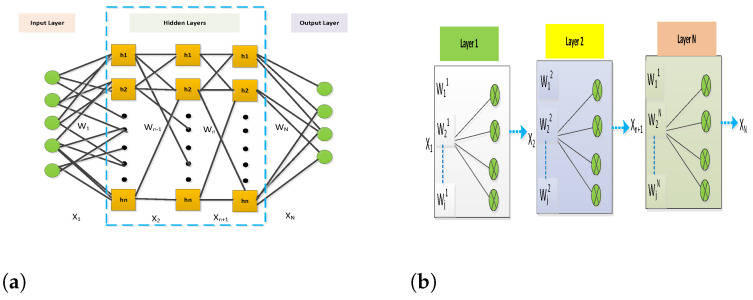
VCSEL-based ML model. (**a**). Fully connected deep neural network description (N layers), including input vectors; (**b**) representation of activation and matrix vector in each layer.

The advancement in ML algorithms and growth in computing power has emerged an optical neural network (ONN) mechanism to utilize the ML applications in the optical domain for high data and low-cost multi-channel transmission. Figure 1a presents a fully connected deep neural network containing N layers. Each layer of N layers includes activation function $f_{NL}\left(.\right)$ and matrix vector multiplication $X_{1\times n}^{i}W_{n\times m}^{i}=Z_{1\times m}^{i}$ as declared in Figure 1b. The input vector $X_{1\times n}$ is encoded in different time steps $t_{s}$ to the phase or amplitude of the laser oscillator with output beams *m* used for parallel processing. The $W_{n\times m}^{i}$ (weight matrix) is mapped in $t_{s}$ with phase encoding to *m* transmitters using $sin\left[\phi_{W_{m}}\left(t\right)\right]\alpha W_{nm}$.

The homodyne product is produced among laser fields by beating each weighting laser on the photo receiver. The achieved photocurrent over $t_{s}$ is measured as

(1) $$I_{m}=\sum_{n}B_{W_{nm}B_{X_{n}}}sin\left[\phi_{W_{nm}-\phi_{X_{n}}}\right]$$

where the amplitude of weight and input lasers are denoted by $B_{W_{nm}}$ and $B_{X_{n}}$, respectively, while the terms $\phi_{W}nm$ and $\phi_{X}n$ are used for the phase of weight and input lasers. The linear multiplication of amplitude-encoded input data $B_{X}\left(t\right)\alpha X_{n}^{i}$ is written as

(2) $$f_{L}\left(.\right)\alpha B_{X}\left(t\right)sin\left[\phi_{W}\left(t\right)\right]=X_{n}W_{nm}$$

The nonlinear multiplication with phase encoded sin[$\phi_{X_{n}\left(t\right)}\alpha X_{n}^{i}$] is calculated as

(3) $$f_{NL}\left(.\right)\alpha sin\left[\phi_{W}\left(t\right)-\phi_{X}\left(t\right)\right]=W_{nm}\sqrt{1-X_{n}^{2}}-X_{n}\sqrt{1-W_{nm}^{2}}$$

**Figure 2:**
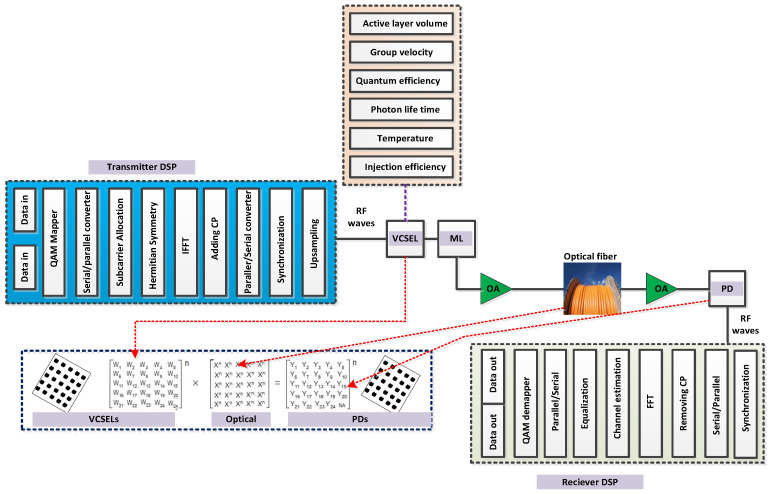
OWN structure using VCSEL-based ML for high data rate and multi-channel transmission.

The properties such as the high directional beam and the narrow band of the VCSEL array have made for a fruitful optical source for high-capacity OWN. In this paper, the suggested VCSEL-based ML optical source is applied to the proposed OWN as depicted in Figure 2. The VCSEL attains data input data from the transmitter-based digital signal processing (DSP) block, including all the required procedures. The carrier number and photon density of transmitted optical signal is estimated [21,22,23] as

(4) $$\frac{dC(t)}{dt}=\frac{\eta_{in}I}{q.U}-\frac{C(t)}{\tau_{e}}-g\left(t\right)\vartheta_{gr}\delta \left(t\right)$$

where δ(t) is the photon density, C(t) is the carrier number, $\eta_{in}$ is the injection efficiency, *U* is the volume, *q* is the charge, *I* denotes the current density, the group velocity is denoted by $\vartheta_{gr}$, g(t) is the gain coefficient, and $\tau_{e}$ is the electron life time. The gain coefficient g(t) is further described as

(5) $$g\left(t\right)=\zeta_{o}\frac{C(t)-C(i)}{1+\xi \delta (t)}$$

where ξ describes the gain saturation coefficient, the carrier density at transparency is denoted by C(i), and $\zeta_{o}$ presents the linear gain coefficient. The photon density is time-dependent, and its derivative is calculated as

(6) $$\frac{d\delta (t)}{dt}=g\left(t\right)\vartheta_{gr}\delta \left(t\right)+K\iota_{sp}\iota_{re}-\frac{\delta (t)}{\tau_{p}}$$

where $\iota_{sp}$ and $\iota_{re}$ are the spontaneous emission factor and recombination ratio, respectively; *K* is the confinement factor; and $\tau_{p}$ represents the photon lifetime. The photon density can be derived from the photon density rate equation in terms of the threshold current, which is given [24,25] as

(7) $$\delta =\frac{\eta_{in}\left(I-I_{tsh}\right)}{qug_{tsh}\vartheta_{gr}}$$

The output power of the laser is evaluated by using threshold current $I_{tsh}$, the mirror loss $l_{m}$, and the internal loss $l_{i}$, given [26,27] as

(8) $$p_{o}=\eta_{in}\frac{l_{m}h\nu }{l_{m}+l_{i}q}\left(I-T_{tsh}\right)$$

In order to briefly describe the transmitter DSP of proposed OWN (Figure 2), the pseudo random binary sequence (PRBS) generates the data sequence for carrying channel information. This information is then mapped into the quadrature amplitude modulation (QAM) format and passed over serial to parallel (S/P) conversion. In the next process, the signals are loaded on a subcarrier of orthogonal frequency division multiplexing (OFDM) signals. The 120 data subcarrier is used by Hermitian symmetry for loading QAM signals. To realize the OFDM modulation, the inverse fast Fourier transform (IFFT) procedure is executed with 256 points. To complete the OFDM symbols and minimize the inter symbol interference (ISI), the cycle prefix (CP) is inserted, and then parallel to serial (P/S) conversion is applied to attain the serial form of OFDM achieved signals. In the final step, the QAM-OFDM signal is passed over the synchronization block, purposing to upsample the QAM-OFDM signals. The AWG7000A waveform generator is then implemented to induce the optical signal and convert the upsample signals into a continuous form. Furthermore, to emit the light of wavelength for optical propagation channels, the ML-based VCSEL is applied. At the receiver side, Rx, the DSP block is performed after photodetector (PD), to recover the transmitted information. The number of elements, with their sizes, installed for evaluating VCSEL-based OWN, is listed in Table 1. Besides that, to estimate the VCSEL-based OWN-performance-based laser RIN, threshold current, main noise, and receiver thermal noise, the BER is described as

(9) $$BER=\frac{1}{4}\left[erf\left(\frac{I-I_{tsh}}{\varrho \sqrt{2}}\right)+erf\left(\frac{I_{tsh}-I}{\varrho \sqrt{2}}\right)\right]$$

In this paper, the leaky rectified linear (ReLU) activation function is used in the ML model, which is an improved version of the ReLU function. The key feature of this function is it can handle the dying problems that occur in conventional ReLU versions. This advanced mechanism has boosted the performance of the ML process, and it can be used in different fields to improve its performance. In this paper, the features of the Improved ML model are utilized in VCSEL to optimize its energy consumption of VCSEL and improve its SE.

## 3. Results Analysis

The simulation analysis of the proposed VCSEL-based OWN is discussed in terms of various estimating parameters, which are investigated as follows.

### 3.1. Spectral Efficiency Analysis

**Figure 3:**
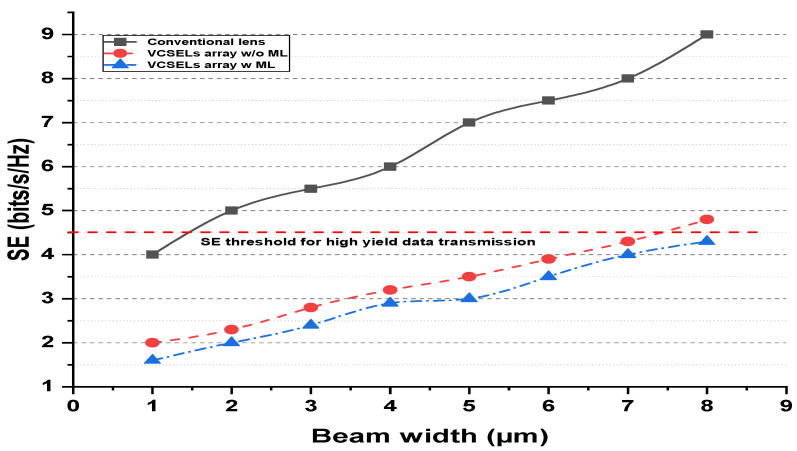
Spectral efficiency vs. beam-width analysis for the conventional laser, VCSEL using ML, and traditional VCSEL.

The beam width of the suggested ML-based VCSEL is analyzed and compared with traditional laser sources using spectral efficiency (SE), as mentioned in Figure 3. The SE is evaluated in bit/s/Hz for three conventional laser sources, VCSEL using ML and VCSEL without ML algorithm. The threshold line is used for calculating the acceptable SE to high-capacity OWN. The OWN needs the spectrum to be efficient, which is defined as

(10) $$SE=\frac{R/B}{k}$$

where *R* is the bit rate, *B* is the bandwidth and *k* is the cluster size. The R/B fraction is considered to link SE. It is seen from Figure 3 that high SE is received by applying a conventional laser source in OWN. In addition, this growth is further upgraded by increasing the beam width range. Thus, the traditional laser source gives the outcome beyond the threshold at 2 μm beam width. In conclusion, the conventional laser source is not applicable for multi-channel and high clock rate data transmission based on OWN. On the other hand, investigating the results attained by ML-based VCSEL and without using ML, it is clarified that ML-based VCSEL has organized SE even at high beam width. As for VCSEL using the ML algorithm, the SE is recorded with significant achievements. However, it fails at high beam width.

### 3.2. Received Power Analysis for ML-Based VCSEL

**Figure 4:**
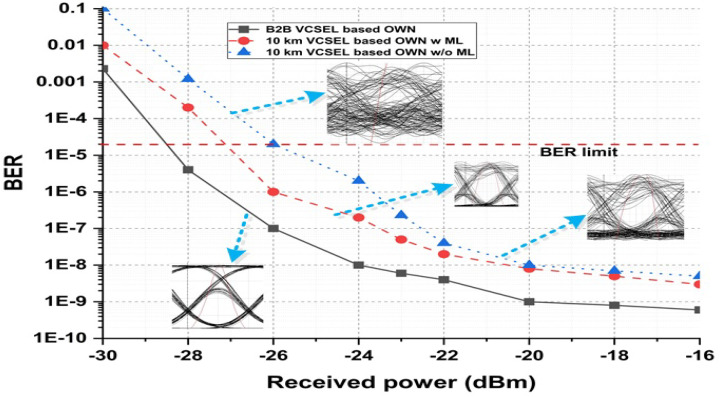
ML-based VCSEL OWN achievements in terms of BER against output power, estimating B2B VCSEL-based OWN, VCSEL-based OWN at 10 km using ML, and traditional VCSEL-based OWN at 10 km.

The presented VCSEL is tested in OWN for discrete power levels as a function of BER, and the results are correlated with a conventional laser source as discussed in Figure 4. The results in Figure 4 are integrated into different power ranges for VCSEL-based back to back (B2B) OWN, VCSEL-based 10 km OWN using ML, and VCSEL-based 10 km OWN without applying the ML algorithm. Figure 4 presents that the outcomes of the B2B-based structure are ideal cases and generate a clear eye diagram, which is shown in the figure with the B2B results line. Comparing the outputs of VCSEL-based OWN with and without using an ML model at a 10 km path range, it is shown in Figure 4 that valuable power can be restored by implementing ML-based VCSEL. The measured results are also analyzed with the help of eye diagrams, which display a significant difference between ML-based VCSEL and simple VCSEL. Investigating the BER limit in Figure 4, it is proved that the ML-based VCSEL gives BER below the threshold limit earlier than using simple VCSEL for high-capacity OWN.

### 3.3. Measuring Result Analysis Using Data Rate and Optical Spectrum Visualizer

**Figure 5:**
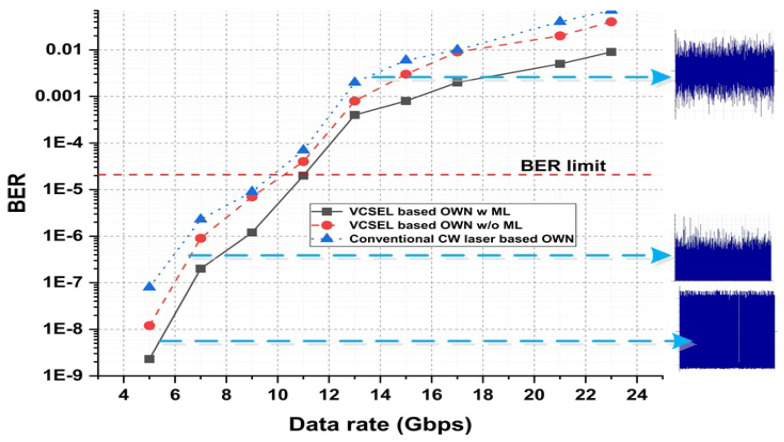
Data rate evaluation as a function of BER using VCSEL-based OWN with ML, conventional laser, and VCSEL-based OWN without ML.

Figure 5 shows the performance of the proposed OWN, related to the data rate and BER. For this study, three types of analysis are performed: (1) VCSEL-based OWN with ML, (2) VCSEL-based OWN without ML algorithm implementation, and (3) conventional laser source-based OWN. It is shown in Figure 5 that the ML-based VCSEL outperforms the plain VCSEL and conventional laser sources. Furthermore, the achievements of all three investigations are compared in terms of the optical visualizer (OV), whose outcomes are mentioned in Figure 5. This explains that the ML-based VCSEL procedure smooths OV by using conventional approaches at high data transmission. The received BER of the conventional-laser-source-based OWN is noted above the threshold line at a low data rate speed (below 10 Gbps). So, for high capacity and data rate OWN, the ML-based VCSEL is a productive solution.

### 3.4. VCSEL-Based OWN Analysis Using Fiber Length and Channel Spacing

**Figure 6:**
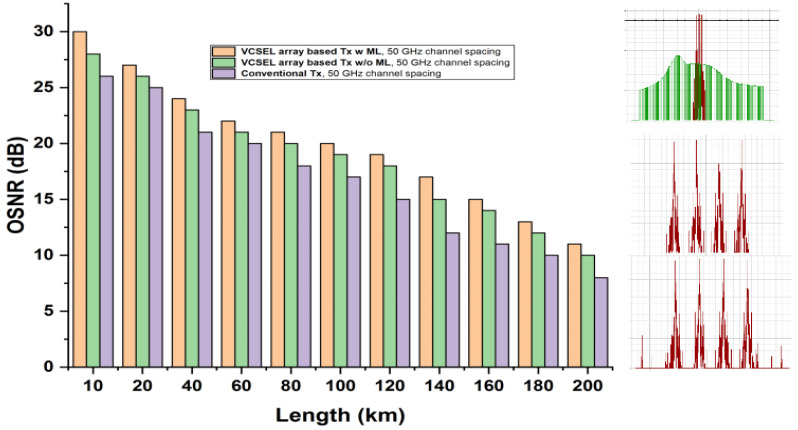
Transmission range performance of proposed OWN vs. OSNR VSEL with ML and without ML at 50 GHz channel spacing.

An optical spectrum analyzer (OSA) is used with 50 GHz channel spacing to analyze the OWN efficiency for divergent fibre length, as shown in Figure 6. The performance of OWN for a diverse range of fiber distances against the optical signal-to-noise ratio (OSNR) is examined, installing ML-based VCSEL, original VCSEL, and conventional laser sources. As it is known that the channel data transmission in OWN with less channel spacing experiences nonlinear issues, and to address the nonlinear problems, a narrow beam with better SE-based laser sources is needed. Hence, in Figure 6, we have reviewed the efficiency of ML-based VCSEL and compared it with traditional laser sources. The outlets declare that the ML-based VCSEL induces light signals with narrow bandwidths, transmitting high-capacity data up to a large coverage area. Adding to the Figure 6 discussion, the OSAs are contrasted among ML-based VCSEL, plain VCSEL, and conventional laser sources, which conclude that conventional laser sources create huge noises.

### 3.5. Polarization, VCSEL Spectrum, and Constellation Visualizer Analysis

**Figure 7:**
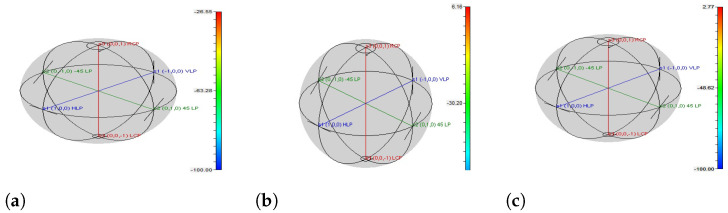
Polarization analyzer; (**a**) conventional laser based OWN outcomes, (**b**) polarization analyzer performance using ML-based VCSEL for OWN, and (**c**) VCSEL-based OWN results with using ML technique.

**Figure 8:**
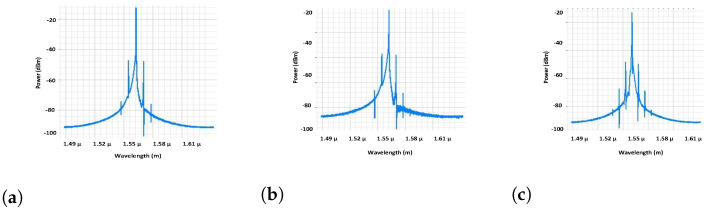
VCSEL spectrum analyzer. (**a**) ML-based VCSEL, (**b**) conventional model, and (**c**) simple VCSEL.

**Figure 9:**
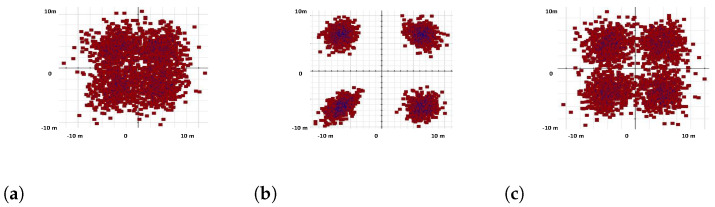
Constellation-diagrams; (**a**) constellation visualizer outputs with conventional laser, (**b**) constellation visualizer results using ML-based VCSEL, and (**c**) constellation visualizer outcomes using simple VCSEL.

In this paper, the proposed OWN and VCSEL light source products are evaluated using distinct visualizers such as the polarization visualizer, VCSEL visualizer, and constellation visualizer. These estimations are displayed in Figure 7, Figure 8 and Figure 9, where Figure 7a shows that ordinary laser sources based on OWN outputs contain large phase differences, which distort the OWN channel information at the receiver side. On the other hand, Figure 7b shows that by employing ML-based VCSEL, the phase change is deduced from 62 to 30 deg. This phase variation is increased again when we use simple VCSEL in the OWN model; however, this change is not greater than in the standard laser sources. Similarly, Figure 8a–c match the VCSEL spectrums of ML-based VCSEL, plain VCSEL, and conventional VCSEL, which depict that the normal laser source has worse outlets than ML-based VCSEL and plain VCSEL. The constellation visualizer analysis is practised in Figure 9a–c, which describes the unsatisfactory spectrum of common laser sources as correlated with ML-based VCSEL and original VCSEL. Thus, the measuring investigation of Figure 7, Figure 8 and Figure 9 summarize that for high capacity and data rate OWN and to support 5G and 6G services, the ML-based VCSEL is a practical solution, aiming to induce a high wavelength for carrying channel information over OWN.

## 4. Conclusions

The highlighted goals of the current communication system are to support 5G and 6G demands and maintain OWN performance. In this paper, the version of VCSEL-based is modified through an ML algorithm and applied in OWN for the first time. We also investigated the QAM-OFDM-based transmitter DSP to update OWN capacity and transmission speed further. The mathematical background and ML algorithm are formulated in detail, including the input and output matrices. The measuring analysis concluded that ML-based VCSEL generates a narrow spectrum of light wavelength compared to the conventional models. The simulation estimations are performed in terms of the power levels; fiber range; BER; RIN; and diverse visualizers such as the constellation visualizer, the polarization visualizer, the optical spectrum visualizer, and the VCSEL spectrum visualizer, which demonstrate the reliable and efficient achievements of ML-based VCSEL OWN in parallel with existing methods. As this mechanism is considered low-cost and less complex for handling 5G and 6G services, we can utilize advanced ML algorithms to obtain massive MIMO-based OWN in the future.

## Figures and Tables

**Table 1:** ML-based VCSEL and OWN parameter list for investigating system reliability.

Parameter	Units
IFFT size	256
Length of training sequence	2
Fiber length	10 to 100 km
QAM order	4
cycle prefix	1/32
VCSEL wavelength	850 to 1300 nm
Responsivity	0.4 A/W
Area of photodetector	20 mm^2^
Current spectral density	4 pA$\sqrt{Hz}$
Bias current	9 mA

## Data Availability

The data will be available as per request.
